# Slow development of woodland vegetation and bird communities during 33 years of passive rewilding in open farmland

**DOI:** 10.1371/journal.pone.0277545

**Published:** 2022-11-11

**Authors:** Richard K. Broughton, James M. Bullock, Charles George, France Gerard, Marta Maziarz, Wesley E. Payne, Paul A. Scholefield, Daniel Wade, Richard F. Pywell

**Affiliations:** 1 UK Centre for Ecology & Hydrology, Wallingford, Oxfordshire, United Kingdom; 2 Department of Biology, University of Oxford, Oxford, United Kingdom; 3 Museum and Institute of Zoology, Polish Academy of Sciences, Warsaw, Poland; 4 Department of Biological and Marine Sciences, University of Hull, Hull, United Kingdom; 5 UK Centre for Ecology & Hydrology, Lancaster Environment Centre, Bailrigg, Lancaster, United Kingdom; USDA Forest Service Southern Research Station, UNITED STATES

## Abstract

Passive rewilding is a potential tool for expanding woodland cover and restoring biodiversity by abandoning land management and allowing natural vegetation succession to occur. Land can be abandoned to passive rewilding deliberately or due to socio-economic change. Despite abandonment being a major driver of land use change, few have studied the long-term outcomes for vegetation and biodiversity in Western Europe. Studies are also biased towards sites that are close to seed sources and favourable to woodland colonisation. In this case-study, we reconstruct a time series of passive rewilding over 33 years on 25 ha of former farmland that had been subject to soil tipping, far from woodland seed sources. Natural colonisation by shrubs and trees was surveyed at three points during the time series, using field mapping and lidar. Breeding birds were surveyed at three time points, and compared with surveys from nearby farmland. Results showed that natural colonisation of woody vegetation was slow, with open grassland dominating the old fields for two decades, and small wetlands developing spontaneously. After 33 years, thorny shrub thickets covered 53% of the site and former hedgerows became subsumed or degraded, but trees remained scarce. However, the resulting habitat mosaic of shrubland, grassland and wetland supported a locally distinctive bird community. Farmland bird species declined as passive rewilding progressed, but this was countered by relatively more wetland birds and an increase in woodland birds, particularly songbirds, compared to nearby farmland. Alongside biodiversity benefits, shrubland establishment by passive rewilding could potentially provide ecosystem services via abundant blossom resources for pollinators, and recreation and berry-gathering opportunities for people. Although closed-canopy woodland remained a distant prospect even after 33 years, the habitat mosaic arising from passive rewilding could be considered a valuable outcome, which could contribute to nature recovery and provision of ecosystem services.

## Introduction

Rewilding is conceived as an approach to restoring dynamic ecosystems that are regulated by natural processes and largely free from human management, but also incorporating the needs of stakeholders and wider society [1, 2]. ‘Passive’ rewilding occurs when habitats and ecosystems are allowed to develop by natural succession in the absence of direct human intervention, such as on abandoned land. The hope is that this approach restores processes to include disturbance regimes, trophic complexity and species turnover, and this is more important than achieving a pre-determined target ecosystem [1–3].

Passive rewilding can occur when farmland, industrial or other land becomes abandoned due to economic or social changes [4, 5]. Increasingly, however, land may be deliberately taken out of management and abandoned for passive rewilding in order to restore habitats and biodiversity, increase ecosystem services and resilience, and for scientific research [2, 6–8].

Although rewilding has been framed as not aiming for a target ecosystem [9], there is growing interest in passive rewilding as a mechanism for woodland creation. Fostering the natural colonisation of woodland on abandoned land has been promoted to expand native forest, restore habitats for nature recovery and provide ecosystem services, such as carbon sequestration and water regulation [10–13]. As a means of expanding woodland, advantages of passive rewilding over tree-planting can include prevalence of genotypes with adaptation to local climate and soils, avoiding imported saplings that may introduce pests and diseases, and reduced management costs, although the outcomes are inherently less predictable [14, 15].

Yet, despite abandonment being one of the major drivers of land use change in Europe [4], there are few well-documented, long-term examples of the outcomes of passive rewilding in this region, particularly from the intensively farmed landscapes that cover much of temperate Western Europe. This is despite studies being urgently needed to inform future land-use policy [4, 16]. Long-term studies have rarely been planned in advance, and so time series data is often incomplete or opportunistic [8, 17].

The limited information from Western Europe, in addition to the more extensive literature from global studies [3, 8, 18], indicates that the speed and manner of woody vegetation succession is most influenced by climate, soil type, former land use, proximity of woodland seed sources and browsing pressure from herbivores. Generally, sites with disturbed ground, close to abundant seed sources in surrounding forest, and with low densities of herbivores (especially deer and other ungulates) can rapidly progress to closed-canopy woodland within several decades [3, 8]. However, published studies are reported to be biased towards those showing successful colonisation of woodland, with relatively few examples of the trajectory of passive rewilding on sites that are considered to have less favourable characteristics [18, 19].

Successful natural colonisation of woodland on former farmland can have mixed outcomes for ecosystems and the services they provide; that is, ‘win-wins’ are rare for land use change [20]. Due to habitat changes and intensification, many European farmland and woodland birds have shown strong population declines in recent decades [21], particularly in Britain [22]. However, increasing tree cover on abandoned land can result in characteristic farmland species being replaced by woodland species as natural colonisation progresses [23, 24]. Land-use change from farmland to woodland can also result in loss of cultural landscape features, such as hedgerows, and associated traditions [4]. Nevertheless, expanding or restoring woody habitats in intensively farmed landscapes can provide important habitat refuges for some rare bird species [5] and may support a greater regional biodiversity [7, 25, 26]. However, there is little information for how passive rewilding may influence species communities in the long term. Nevertheless, important ecosystem services resulting from a transition to increased woodland cover and reduced soil disturbance can include lower flood risk and improved carbon storage [10]. As such, passive rewilding of former farmland has a potentially important role in the restoration of species, habitats and wilder landscapes, and could help to meet targets for woodland expansion, nature recovery and climate action [6, 10].

Quantifying natural colonisation towards woodland can be challenging, due to issues of scale and vegetation complexity, and studies have typically been limited to broad-scale mapping at low resolution, or detailed sampling of smaller plots [27, 28]. More precise remote sensing tools, such as lidar (light detection and ranging), can overcome many of these limitations by characterising the height and extent of woody vegetation at high resolution across entire landscapes [29]. Lidar has enabled time-series or chronosequence inventories of woodland colonisation of former farmland in unprecedented detail and completeness [8, 30]. Nevertheless, such studies remain uncommon.

The aims of this study are to assess the long-term natural colonisation of woody vegetation on an abandoned farmland site, far from woodland seed sources. Using complementary data collection of airborne lidar and field surveys, we reconstruct a time series to quantify the natural colonisation of tree and shrub vegetation over three decades of passive rewilding, including the fate of pre-existing hedgerows. This unique case study is complementary to that of Broughton et al. [8], which used a similar approach to show rapid colonisation of abandoned farmland on a contrasting site, adjacent to mature woodland and abundant seed sources. Additionally, we quantify parallel changes in the bird community on this site over the same period, and compare this with the bird community of intensively managed farmland in the wider region as a counterfactual. Our main hypotheses were that woody colonisation would occur slowly, but would be reflected by parallel changes in the bird community, particularly an increase in species associated with wooded habitats. We discuss the results in relation to policies for woodland expansion, restoring biodiversity and enhancing ecosystem services.

## Materials and methods

### Study area

The study site was the Noddle Hill Local Nature Reserve (hereafter: Noddle Hill) on the outskirts of the city of Kingston upon Hull in northeast England (53° 48′N, 0° 18′W). Noddle Hill falls within low-lying floodplains of the tidal River Hull and Humber estuary, with lime-rich loamy and clayey soils and peaty depressions. High groundwater results in seasonally wet soils and some shallow seasonal flooding, with ground elevations mostly at 0.5–1.5 m above sea level. The temperate climate has mean annual temperatures of 6.7–14.0°C and a mean annual rainfall of 680 mm recorded for 1981–2010 on a weather station 5.8 km away [31].

Noddle Hill is surrounded by intensive arable farmland and some grazing pasture, with amenity sports fields and residential housing to the west, established since the late 1960s. During the 19^th^ and 20^th^ Centuries, Noddle Hill and surrounding farms were mixed dairy cattle and arable, graded as ‘moderate quality’ agricultural land [32]. Grazing pastures were seasonally wet meadows and improved grassland sown with clover [33].

The current study focuses on seven contiguous fields, totalling 25.0 ha, during 1988–2021. The fields were grazed by cattle until about 1980 and then left fallow. In 1988, clayey soils from elsewhere in the city were dumped over 69% of the site to an average additional depth of around 1.0 m, increasing the mean ground elevation to 2.1 m (maximum 5.8 m). The aim of dumping was landscaping for future development, but the site was abandoned before completion, with natural succession of the vegetation taking over. As such, the initial ground surface for rewilding was a patchwork of bare soil, seasonally wet grassland or ex-arable, and the original 4.9 km of field-boundaries comprising drainage ditches and 2.7 km of hedgerows dominated by common hawthorn *Crataegus monogyna*. Adjacent old fields (8.7 ha) were subject to tree-planting and landscaping from 2000, and we excluded them from the study. These planted trees were too immature to act as a seed source during the study.

### Local seed sources

Over the 33 year duration (1988–2021), woodland seed sources remained locally rare. In 1988, only a single mature crack willow *Salix fragilis* was present on Noddle Hill, along with young crack willows, fruiting common hawthorns and bramble *Rubus fruticosus* in the hedgerow network (RKB pers. obs.). During the study period, the surrounding area within 1 km distance from the site boundary contained only 5.3 ha of mature woodland (1.0% cover) as small copses or shelter belts of broadleaved trees. A further 23.9 ha of young trees was planted from 2000, but remained too young to act as seed sources during the study. The nearest significant mature woodland (10.8 ha) is 1.5 km distant. Mature local trees are dominated by common ash *Fraxinus excelsior*, pedunculate oak *Quercus robur* and sycamore *Acer pseudoplatanus*. Approximately 11 km of hedgerows and linear woody features fall within the 1 km radius, dominated by common hawthorn.

Other mature trees and shrubs currently found within 1 km of Noddle Hill, which could act as seed sources, include common sallow *Salix cinerea*, dog rose *Rosa canina*, European elder *Sambucus nigra* and blackthorn *Prunus spinosa*, with occasional European beech *Fagus sylvatica*, hybrid black poplar *Populus x euramericana*, black alder *Alnus glutinosa*, Scots pine *Pinus sylvestris*, goat willow *Salix caprea*, field rose *Rosa arvensis* and crab apple *Malus sylvestris*.

Many of the species listed above are wind dispersed, but pedunculate oak, rose spp., blackthorn, crab apple and bramble produce fruits that are attractive to a range of animals that can disperse them. Potential animal dispersers at Noddle Hill include abundant berry-eating thrushes (Turdidae) and seed-hoarding wood mice *Apodemus sylvaticus*. Some important potential dispersers of large tree seeds (e.g. acorns) were rare or absent, notably grey squirrels *Sciurus carolinensis* (colonised in the 1990s but remained rare), and Eurasian jays *Garrulus glandarius*, which are not resident in the region [34, 35].

### Herbivores and vegetation disturbance

Herbivores were generally scarce throughout the study period [34]. European rabbits *Oryctolagus cuniculus* and brown hares *Lepus europaeus* were uncommon during site visits, but roe deer *Capreolus capreolus* colonised during the study and up to five were recorded by 2021 (i.e. 1 deer per 5 ha). Intermittent, localised grazing by 3–4 tethered ponies occurred on part of the site from around 2001–2007. However, the overall impact of ponies on vegetation development was likely to be low or negligible, due to its limited extent and duration.

As such, in addition to the very limited seed sources and dispersal agents for natural colonisation (particularly for large-seeded trees) that are outlined above, Noddle Hill is also characterised by little grazing pressure or other significant impediments to woody vegetation succession over the 33 year duration of study.

### Habitat data

The vegetation development since 1988 was documented in three time periods, between four and 33 years after abandonment (Table 1). This involved a combination of field surveys for site mapping and sample plots, and, for the two later periods, remote sensing surveys using lidar. The aim was to determine the progression of woody vegetation colonisation over time, including what happened to the existing hedgerows, and which broad habitats had established by the end of the study period.

**Table 1:** Timing of data collection during three survey periods for bird, lidar and vegetation field surveys at Noddle Hill (NH), with bird data from Breeding Bird Surveys (BBS, a national monitoring program) on regional farmland for comparison. Abandonment and passive rewilding began in 1988.

Survey period	Vegetation field survey	Lidar	NH bird survey	BBS bird survey
1	1992	none	1992	1994
2	2001	2002	2006	2006
3	2021	2021	2021	2017

### Field surveys of woody vegetation and broad habitats

The first field survey was in August 1992 after four years of rewilding. The full site was mapped during a detailed walkover survey to record and map broad habitat features in the manner of a standard ‘phase 1 habitat survey’ [36], including hedgerows, mature trees, wetlands (ponds and seasonally or permanently wet hollows) and the composition of old fields. Mapping was manually ‘by eye’ (i.e. approximately) based on a 1:25000 Ordnance Survey reference of field boundaries. GPS equipment was unavailable at the time.

The second field survey was in August 2001, after 13 years of rewilding, during a formal phase 1 habitat survey by Holloway [37]. The survey mapped the broad habitats and vegetation communities of the old fields, based on a walkover survey, comparable with the 1992 mapping. The 1992 and 2001 maps were digitised in ArcGIS 10.6 (ESRI, Redlands, USA) to estimate the spatial extent of habitat features.

**Fig 1:**
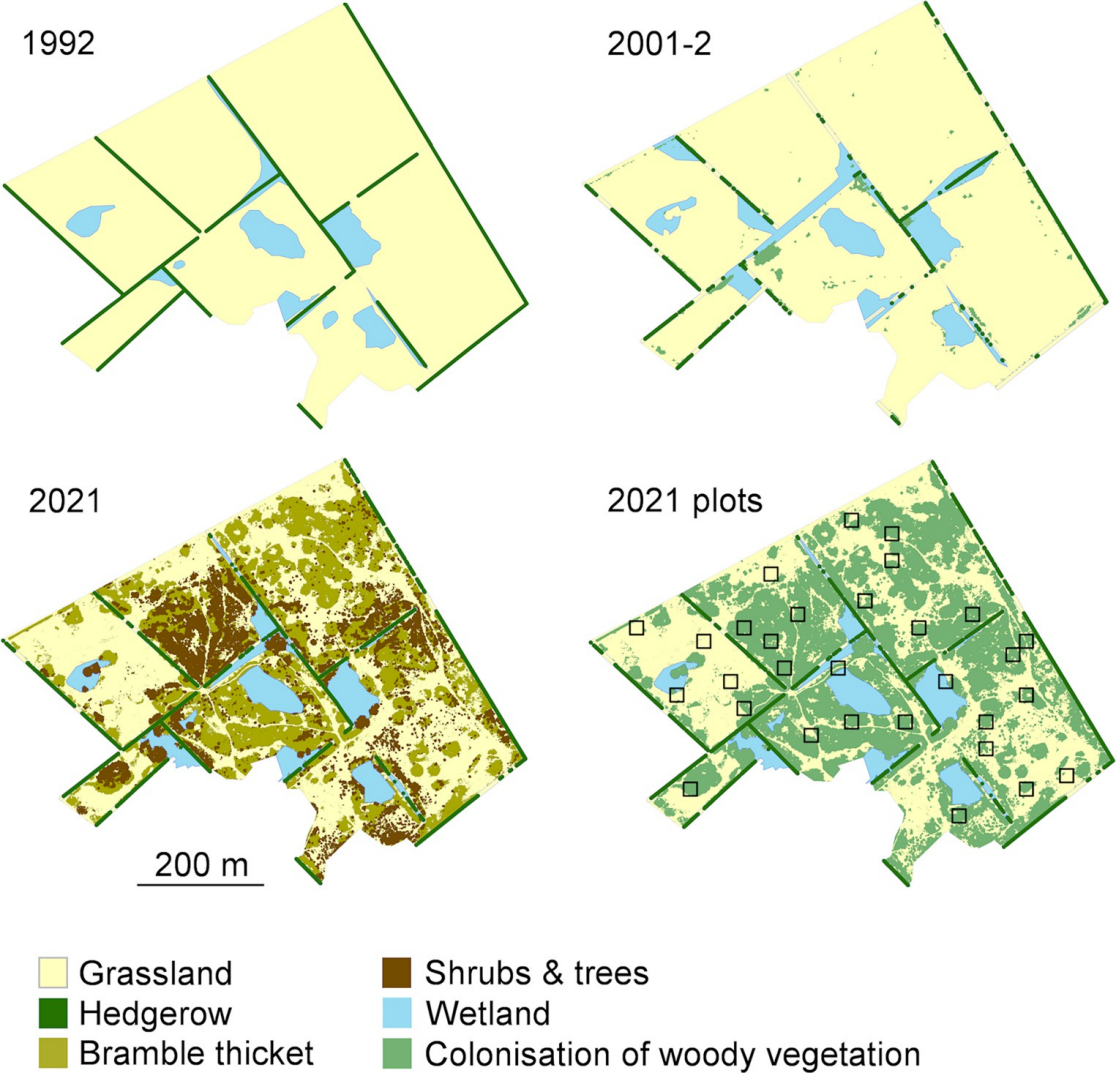
Vegetation and broad habitat change at Noddle Hill during passive rewilding since abandonment in 1988. In 2021 the natural colonisation of woody vegetation on the old fields could be distinguished as either bramble thicket or shrubs and trees. The 30 woody vegetation survey plots in 2021 are also shown (black squares) in relation to the coverage of colonising woody vegetation.

The third vegetation field survey was in July-September 2021, after 33 years of rewilding, and comprised 30 sample plots of 20 x 20 m (aligned on a north axis) to record shrub and tree vegetation. Survey plots covered 5% of the site and were positioned in areas of differing woody vegetation cover to sample the species composition in each old field (Fig 1). Survey plots were marked on the ground using a handheld GPS and measuring tape.

Data collected in survey plots included the number of trees and shrubs of each species that were at least 0.5 m tall. The canopy cover of each species in each sample plot was estimated by eye in increments of 1% up to 5% cover, and thereafter in 5% increments. The diameter at breast height (DBH, at 1.3 m) of each tree was also recorded where access was possible. Bramble was treated as woody vegetation and recorded as thicket cover.

### Lidar vegetation surveys

The vegetation surveys in the second and third time periods included lidar mapping of the woody vegetation extent and height over the whole site (Table 1). For the second period, a digital surface model (DSM), depicting above-ground feature heights, and a digital terrain model (DTM), depicting the ground elevation, were available for December 2002 at 2 m horizontal resolution and a minimum ±0.15 m vertical accuracy from England’s Environment Agency airborne lidar data collection [38]. Subtracting the DTM from the DSM produced a 2002 canopy height model (CHM) of relative height values of vegetation across the Noddle Hill site. However, as the 2002 DSM was captured during leaf-off conditions it was not optimised for accurate vegetation height observations. As such, the 2002 CHM was used to map the presence of woody features, such as hedgerows, bushes and trees, but not to depict heights.

In the third survey period, a drone (UAV) mounted lidar survey was conducted during leaf-on conditions in September 2021, and the data were processed to generate a 0.2 m resolution DSM. The leaf-on conditions meant that the lidar penetration of dense thickets was insufficient to produce an accurate DTM, and so instead we used a 0.5 m Environment Agency DTM derived from airborne lidar acquired during leaf-off conditions in January 2008. This 2008 DTM was subtracted from the 2021 DSM to create a 0.5 m resolution 2021 CHM for mapping woody vegetation extent and to estimate height. Details of lidar data and acquisition are given in S1 Appendix and S1 Dataset.

For the 2002 and 2021 CHMs, height values below 0.5 m were discarded to exclude grasses/herbs, with taller height values considered to be shrubs and trees (as per Broughton et al. [8]). A 3 m buffer on either side of the field boundaries was delineated as the hedgerow network, encompassing linear shrubs as depicted in the 1992 field map. Those parts of the old fields located outside of this hedgerow network were classed as infield areas (23.4 ha).

### Canopy delineation model

To identify individual tree and shrub canopy extents, a canopy delineation model was derived from the 2021 CHM, which was processed using the ForestTools library [39] in the R statistical package. This processing estimated the canopy extents using watershed segmentation. Tree points, canopy height and area statistics were generated as shapefiles in a GIS, covering the whole study area. Dominant treetops were detected using a variable window filter algorithm [40]. The outputs of this process were a tree-crown polygon layer, depicting individual tree and shrub canopies as discrete polygons, and a tree-top point layer that marked the apex of each tree/shrub.

The model can only delineate canopies with an obvious apex, picking out upright shrubs and trees, but low bramble thickets with no obvious apex cannot be delineated. As such, the canopy delineation model is effectively a shrub-tree model, which distinguishes the shrubs and the trees from the woody bramble. By subtracting the shrub-tree model from the original CHM (i.e. all woody vegetation ≥0.5 m tall) we were able to isolate the bramble-dominated thickets to map them separately. The final result is a shrub-tree model and a separate bramble model, allowing a more detailed woody vegetation inventory for 2021.

### Wetlands

The digitised wetlands from the 1992 and 2001 site maps were used to estimate their number and approximate extents, with visible hollows in the 2002 lidar data used for validation. For 2021, wetland extents were again digitised using a walkover field survey to validate features (ponds, wet hollows etc.) visible in the 2021 lidar and ortho-imagery captured alongside. Wetland number and approximate extent were then compared across the three survey periods to assess habitat development as rewilding progressed.

### Noddle Hill bird community

Noddle Hill’s breeding bird community was surveyed during the same periods as the vegetation, i.e. at three points between four and 33 years after abandonment (Table 1). Bird surveys in the first two periods were available from historical monitoring, and in the third period from a new survey following the same methodology. Limited data availability resulted in a 4–5 year time gap between some bird and vegetation surveys (Table 1), but this is considered acceptable in relation to the vegetation’s rate of change.

Each bird survey involved four visits during a spring breeding season (April-June) following a 2.0 km transect route that intersected each old field in the study area. The transect route was the same in the first two periods but modified in 2021 due to inaccessibility as a result of vegetation growth, although it remained 2.0 km long and intersected the same old fields.

All birds detected along the transect route (and variably detectable for approximately 100 m either side) were recorded, including territorial and breeding behaviour (e.g. pairs, singing, feeding young). Double counting was avoided by noting counter-singing and movements during observation. We excluded birds that were flying over and clearly not using the site for foraging or breeding.

### Regional bird community

Baseline bird data from Noddle Hill were not available from before abandonment and rewilding began in 1988. Therefore, to indicate the effect of passive rewilding on the Noddle Hill bird community, and control for other local trends (e.g. weather), we used the bird community of farmland in the local region throughout the study period as a comparator.

Regional bird data were available from the British Trust for Ornithology’s Breeding Bird Survey (BBS), which provides national monitoring of species richness and abundance data in 1 x 1 km sample squares, chosen by stratified random sampling. Surveyors follow two approximately parallel 1.0 km transects (i.e. 2.0 km per square) through each BBS square and record birds on one early (April-early May) and one late (mid May-June) visit during the breeding season [41].

To take account of any changes in the regional bird community over the study period, due to national population trends [42], BBS data was chosen for 1 km squares that were surveyed near in time (0–4 years) and space (< 10 km) to the Noddle Hill surveys, to provide contemporary comparisons (Table 1). Two BBS squares were used for each survey period, providing similar survey effort (four visits per year) as at Noddle Hill. The selected BBS squares were dominated by mixed farmland typical of the region, i.e. 83–96% arable or grazing. The identity of the two BBS squares differed in each survey period, involving five squares overall.

As with Noddle Hill, birds were excluded from BBS surveys if recorded as flying over and not using the site for breeding or foraging.

### Statistical methods

Spatial modelling and analyses of the woody vegetation of infield areas and hedgerows, using ArcGIS and ForestTools, enabled the extraction of descriptive statistics for the infield areas from the 2002 and 2021 CHM derived woody vegetation extents, and the 2021 height metrics. For 2002 the CHM was used alongside the 2001 field map to determine extents. The 2002 and 2021 woody extents were compared quantitatively to the digitised field maps of 1992.

Hedgerow network height summary statistics were calculated from the 2021 CHM, and hedgerow network length from the 2002 and 2021 CHM. To assess whether the network had persisted without management, both 2002 and 2021 hedgerow lengths were compared with the length obtained from the original 1992 survey.

The 20 x 20 m vegetation sample plots from 2021 provided plot species richness and frequency, and cover and size (DBH) metrics of the trees and shrubs. The bramble and shrub-tree models were validated by correlating with the bramble and shrub-tree cover measured in the 30 sample plots.

Bird data were analysed to determine species richness (*α*-diversity), defined as the total species recorded from all visits in a breeding season, which was used as an indicator of the bird community in each survey period. For BBS squares, we used the total number of species in both squares per survey period. Additionally, we calculated the Jaccard similarity coefficient [43] for each survey period, which represents the proportion of species recorded from Noddle Hill or the BBS squares that were also recorded in both. We expected the Jaccard coefficient to decrease over time as the Noddle Hill bird community diverged from the regional BBS squares as rewilding progressed.

We used species abundance (maximum count of each species on any visit within the survey period) to derive the Shannon-Wiener index to quantify bird diversity between survey periods for Noddle Hill and the BBS squares. We expected the index to remain stable over time for the BBS squares, but to increase at Noddle Hill as rewilding progressed and a greater variety of habitats developed. Due to the differing survey areas (extents), indices for Noddle Hill and BBS data were not compared directly.

Bird species recorded at Noddle Hill and in the BBS squares were classified by their primary habitat associations: farmland, woodland or wetland [42]. At Noddle Hill, we expected the number of woodland species to increase and farmland birds to decline as woody vegetation colonised the site.

## Results

### Vegetation after 4 years of rewilding

The field survey from 1992 identified the infield areas as open grassland, with no discernible colonisation of the old fields by woody vegetation (Table 2, Fig 1). Shrubs and trees were mapped as occurring only along the original 2.7 km of linear hedgerows, although information on hedgerow heights was not available.

**Table 2:** Woody vegetation cover and height values (≥ 0.5 m tall) and wetland cover in the infield area (23.4 ha) and full site (25.0 ha, including the hedgerow network) at Noddle Hill for three survey periods since abandonment to passive rewilding began in 1988. The 2021 woody vegetation cover was further sub-categorised into bramble and shrub-tree cover.

Habitat metric	1992	2001–2	2021
Infield woody vegetation cover %	0	2.2	52.3
Infield woody vegetation mean height (SD)	0	-	2.1 (1.8)
Infield woody vegetation median height	0	-	1.4
Hedgerow mean height (SD)	-	-	2.8 (2.3)
Hedgerow median height	-	-	2.4
Hedgerow length (km)	2.7	1.2	1.9
Infield bramble model cover %	-	-	32.2
Infield shrub-tree model cover %	-	-	20.2
Full site wetland cover %	7.5	9.8	7.1
Full site woody vegetation cover %	-	4.5	53.2

### Vegetation after 13–14 years of rewilding

The 2001 field survey mapped the old fields as open mesotrophic grassland, with only 0.05 ha of contiguous shrub thicket in one field. The grassland communities described by Holloway [37] are given in S2 Appendix. The 2002 lidar survey recorded a 0.5 ha woody vegetation colonisation of the infield area (Table 2, Fig 1), supporting the 2001 field survey findings that show a limited natural colonisation by this stage. The linear hedgerow network depicted in the 2002 lidar CHM was only 44.7% of the original length mapped in 1992 (Table 2).

### Vegetation after 33 years of rewilding

Overall, in 2021, the total tree and shrub cover for the whole site after 33 years of rewilding was 53.2%, including infield (49.0%) and hedgerow vegetation (4.3%). Almost a third (30.1%) of the whole site was covered by bramble-dominated thickets, and almost a fifth (18.9%) by other shrubs and trees (Table 2, Fig 1). Woody colonisation was not uniform across the site and resulted in a patchwork mosaic of thickets, with coverage of the seven individual old fields ranging from 22–67% (mean 51%, SD 15%).

**Fig 2:**
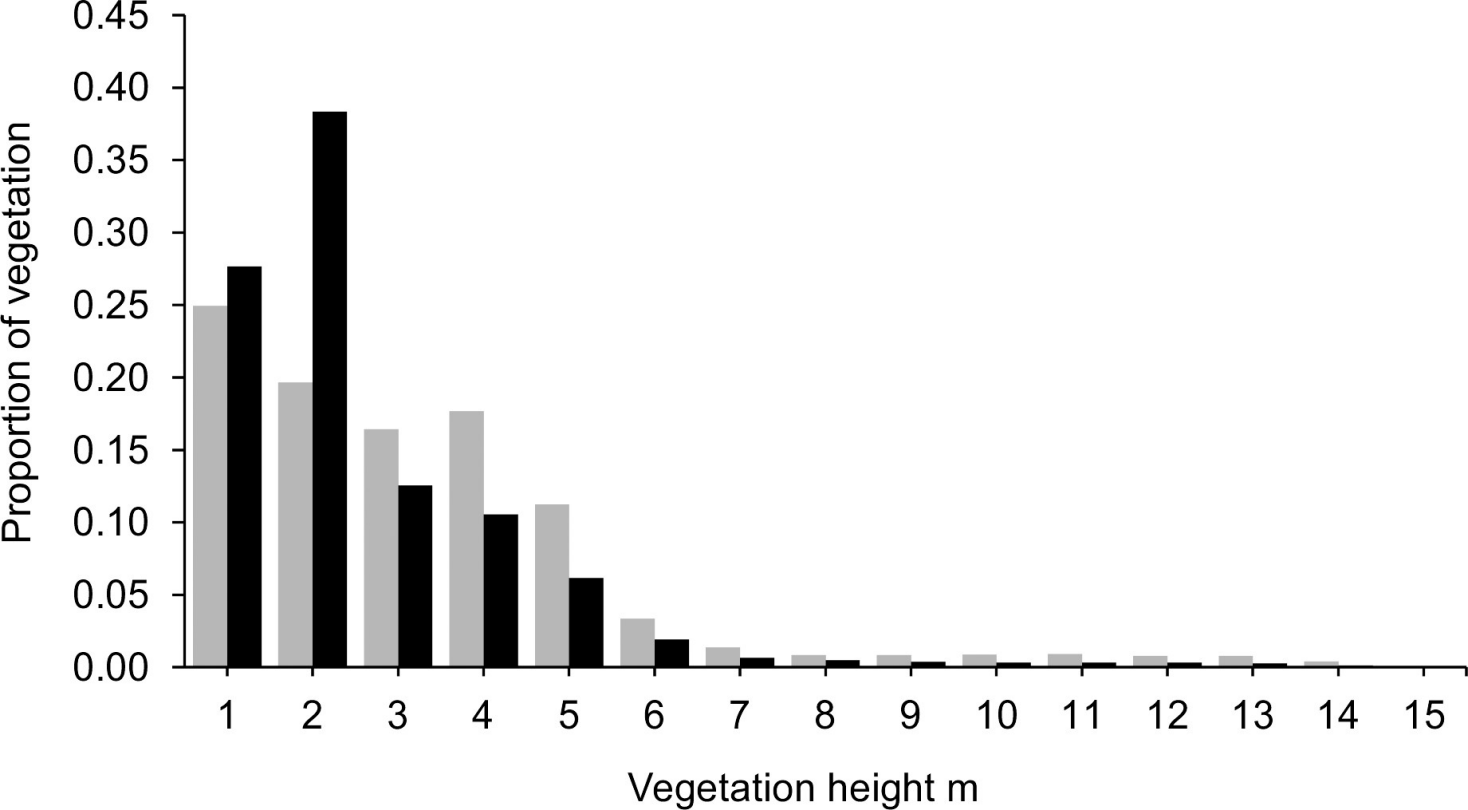
Lidar height profile of woody vegetation in the infield areas (black bars) and the former hedgerow network (grey bars) in 2021, after 33 years of rewilding.

The 2021 lidar survey showed a substantial increase in the natural colonisation of woody vegetation, covering more than half (12.2 ha) of the infield areas (Table 2, Fig 1). The infield woody vegetation height peaks at 1–2 m (Fig 2) and its median and mean heights were less than those of the old hedgerow network, which consisted of older shrubs that pre-dated abandonment (Table 2). Across the whole site, 88% of the hedgerow and infield vegetation was below 4 m tall.

The 2021 shrub-tree canopy delineation model identified 7897 individual shrubs or trees, with 88% in the infield areas (giving a density of 297/ha) and 12% along the original hedgerow network. The shrub-tree model accounted for 38.6% of the woody vegetation cover, with the bramble model accounting for the other 61.4% (Table 2). In the 30 sample plots from 2021 (surveying trees and shrubs at least 0.5 m tall), there was very good correlation between the bramble and shrub-tree canopy cover derived from the model and the field survey (Pearson *r* = 0.80 and *r* = 0.83 respectively), giving reassurance that the classifications were valid.

In these sample plots, woody species richness was low (mean 2.8, SD 1.8, range 0–9), and included the invasive non-native Japanese rose *Rosa rugose* in one plot. The most frequent and extensive woody species were thorny bramble, common hawthorn and rose spp. (Table 3), and small DBH values indicated that the shrubs were mostly immature. Tree species were scarce and were typically young saplings with a small DBH, most frequently common ash and willows/sallows (*Salix* spp.).

**Table 3:** Shrub and tree species metrics in 30 sample plots (20 x 20 m) recorded in 2021, after 33 years of natural colonisation and passive rewilding. Note that the tree/shrub sample sizes for DBH (diameter at breast height) may be lower than the full count due to impaired accessibility.

Species	Frequency % of plots	Count	DBH cm			Cover %	
			Mean	SD	n	mean	SD
Bramble	87.5	-	-	-	-	33.9	27.3
Common hawthorn	75.0	259	6.0	2.8	237	22.2	25.6
Dog/Field rose	46.9	22	-	-	-	0.5	1.0
Common elder	18.8	6	9.3	1.9	4	0.4	1.1
Crack willow	12.5	5	14.4	5.5	4	0.7	2.9
Common ash	9.4	15	7.2	3.3	15	<0.1	-
Pedunculate oak	9.4	4	3.3	1.7	4	<0.1	-
Silver birch	6.3	4	8.1	0.3	4	<0.1	-
Blackthorn	6.3	2	-	-	-	<0.1	-
Grey willow	6.3	11	5.9	1.6	11	<0.1	-
Black alder	3.1	4	6.0	0.0	4	<0.1	-
Field maple	3.1	6	3.7	0.8	6	<0.1	-
Japanese rose	3.1	1	2.0	-	1	<0.1	-

By 2021, woody vegetation along the hedgerow network occupied 71% of its 1992 original length. However, the extent of infield colonisation meant that much of this hedgerow network was subsumed within the shrub thickets (Fig 1).

### Wetlands

**Fig 3:**
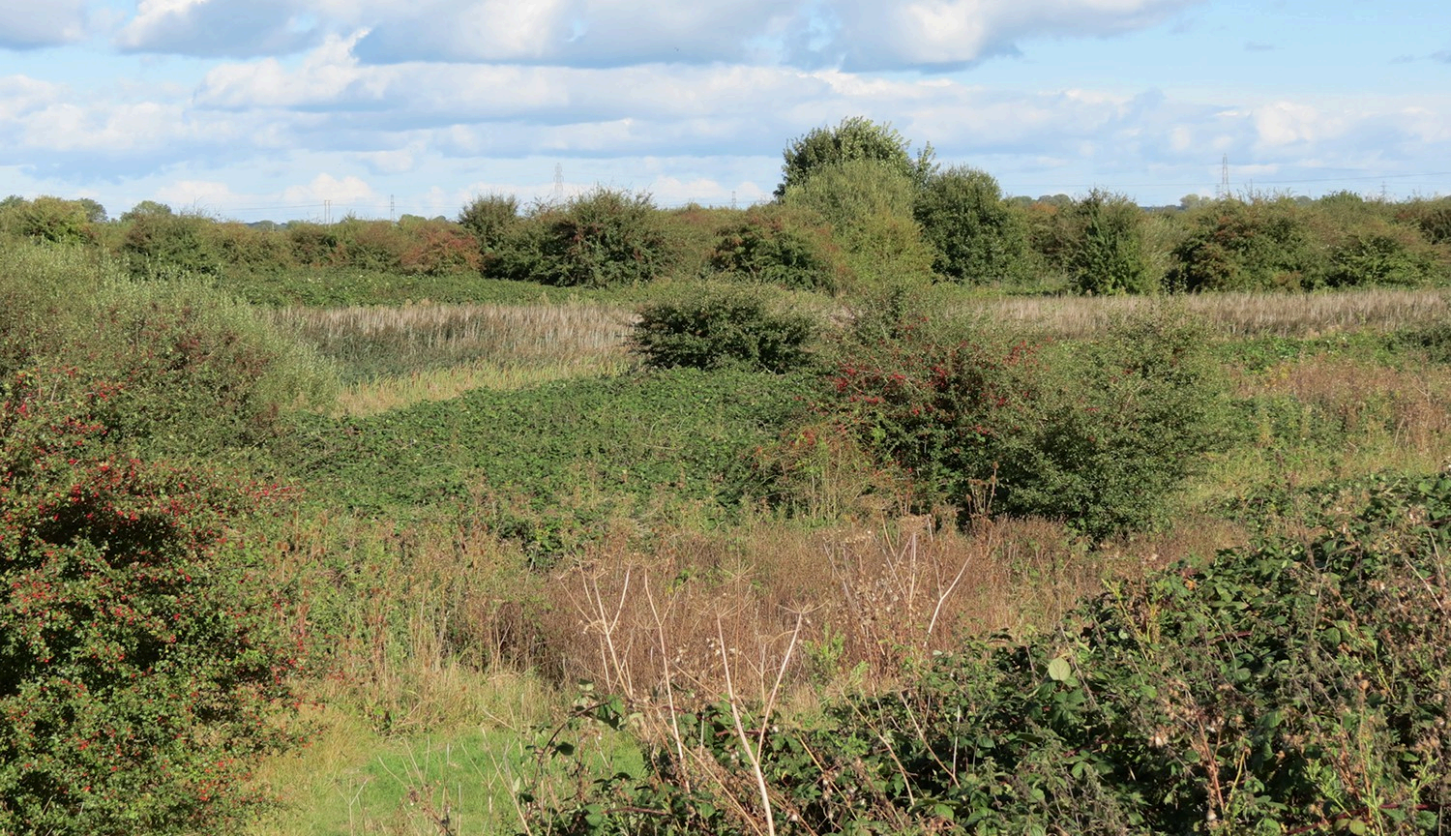
The habitat mosaic at Noddle Hill after 33 years of passive rewilding, showing a shrubland dominated by bramble and common hawthorn thickets, with some open mesotrophic grassland and, in the mid ground, a wetland covered by dense reed-swamp (*Typha*-*Phragmites*-*Glyceria*) vegetation.

Overall, the number, area and location of wetlands at Noddle Hill did not change substantially over the course of the study. The 1992 field survey mapped ten distinct wetlands covering an estimated 1.9 ha (Table 2, Fig 1). Four wetlands were recorded as reed-swamp (*Typha*-*Phragmites*-*Glyceria*) in infield hollows, and the remainder as overflow and expansion from derelict ditches along field boundaries. By 2001–2002, the mapped extent of the ten wetlands covered an estimated 2.5 ha of the site, and all were dominated by reed-swamp vegetation with little open water. The 2021 survey again recorded ten wetlands persisting in similar locations to previous periods, covering 1.8 ha of the site. The wetlands were well vegetated, again as reed-swamp, with negligible open water, and formed part of the shrubland-dominated habitat mosaic (Fig 3).

### Bird communities

The full lists of bird species and abundance counts from Noddle Hill and the BBS squares during each survey period are given in S3 Appendix. Species richness (*α*-diversity) at Noddle Hill and in the regional BBS squares was broadly similar throughout the study periods (Table 4). However, species richness at Noddle Hill declined over the study, but increased slightly in the BBS squares. The Shannon-Wiener indices were also broadly similar for Noddle Hill and the BBS squares, with only a marginal decline for both overall (Table 4). These indices suggested that, despite changes in the bird community at Noddle Hill, the species diversity and evenness of abundances remained relatively constant over time.

**Table 4:** Metrics and indices of the breeding bird community during rewilding at Noddle Hill (NH) and samples from regional farmland in the BTO Breeding Bird Survey (BBS) during a time series of survey periods between 1992 and 2021 (see Table 1).

Bird metric/index	Survey period			Change % over periods 1–3
	1	2	3	
Species richness BBS	39	48	41	+5.1
Species richness NH	48	46	43	-10.4
Jaccard index	0.61	0.62	0.53	-13.1
Shannon-Wiener BBS	3.28	3.58	3.20	-2.4
Shannon-Wiener NH	3.20	3.24	3.14	-1.9

The Jaccard index of similarity showed that Noddle Hill and the BBS squares initially shared most species in the first two survey periods (Table 4). However, by the third survey period, the proportion of shared species declined notably as Noddle Hill diverged from the BBS squares in the wider region.

**Fig 4:**
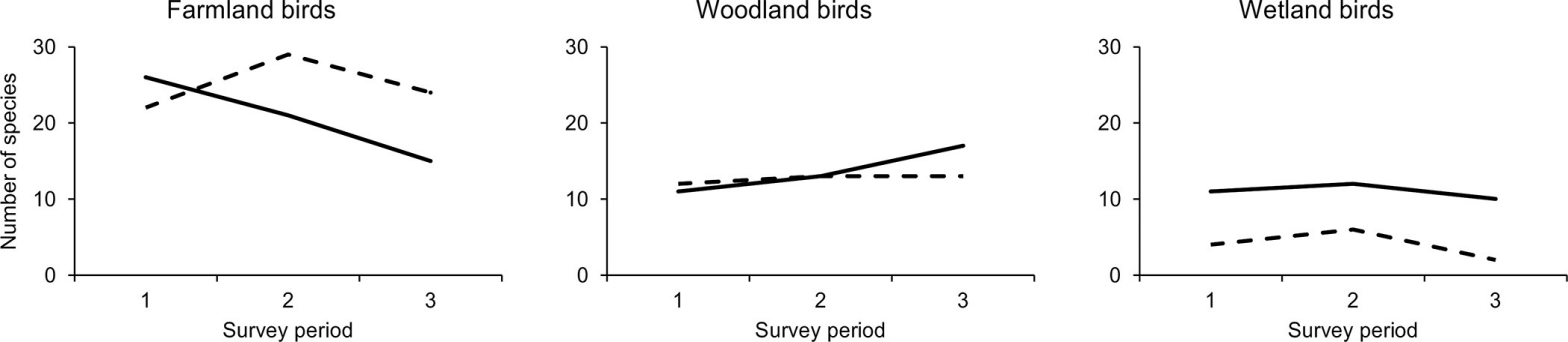
Breeding bird community changes during rewilding at Noddle Hill (solid line) and in regional farmland in the BTO Breeding Bird Survey (BBS, dashed line). Species were categorised according to primary habitat association. Noddle Hill was abandoned to rewilding in 1988 and its three survey periods were 1992, 2006 and 2021, with corresponding periods of 1994, 2006 and 2017 for BBS surveys.

Changes over time in bird species associated with different habitats mirrored changes in those habitats at Noddle Hill. There was a 44.4% decline in farmland bird species over the study period, which was offset by a 54.5% increase in woodland species (Fig 4). Meanwhile, in the BBS squares, the number of farmland and woodland species changed relatively little. Wetland species were also notably more abundant at Noddle Hill throughout the study (Fig 4). As such, by the third survey period, farmland species still dominated the regional bird community in the BBS squares (61.0% of species) but Noddle Hill had changed to comprise 34.9% farmland species, 39.5% woodland species and 23.3% wetland birds (Fig 4).

In particular, major songbird groups were substantially (82.3%) and significantly more abundant at Noddle Hill than in BBS squares by the third survey period, specifically 21 species of warbler (Sylviidae, Acrocephalidae, Cettiidae, Phylloscopidae and Locustellidae), finch (Fringillidae), bunting (Emerizidae) and thrush (Wilcoxon signed rank test: *W* = 39.0, *P* = 0.045; S3 Appendix).

## Discussion

After 33 years of passive rewilding, habitat changes at Noddle Hill (former farmland that was subject to soil tipping) resulted in a mosaic of grassland, wetland and extensive shrub thickets. In the absence of significant nearby woodland or trees to act as seed sources, woody natural colonisation was dominated by thorny shrubs. This shrubland habitat mosaic supported a locally distinctive bird community that contained a relatively high proportion of songbirds and wetland species in particular. As such, although prospects for closed canopy woodland still appear remote after several decades, the rewilding of Noddle Hill has arguably provided biodiversity benefits and potentially other ecosystem services. Indeed, the relatively slow rate of woody vegetation succession has created a complex habitat heterogeneity that may otherwise be difficult to achieve by prescribed habitat creation or woodland planting. Nevertheless, for more rapid creation of native woodland by passive rewilding, the results demonstrate that careful spatial targeting is required.

### Natural colonisation of woody vegetation

The slow natural colonisation of woody vegetation at Noddle Hill reflected the scarcity of local seed sources. This is despite the previous soil tipping over most of the site, which would have been an ideal seedbed for colonising trees and shrubs, and the general lack of medium-large herbivores that would inhibit colonisation and survival [3, 18]. Poor colonisation by trees, in particular, was likely due to seed sources being distant, and further exacerbated by a lack of key seed dispersers, especially Eurasian jays that are important for dispersing acorns of pedunculate oaks to open ground [44].

Mesotrophic grasslands initially developed on the old fields for at least two decades before colonisation by woody vegetation became extensive. After 33 years, dense shrub thickets of thorny bramble, common hawthorn and rose spp. covered more than half of the site, but young trees remained scarce. The few pedunculate oaks that colonised Noddle Hill may have been cached as acorns by rooks *Corvus frugilegus* [45], though most species present are usually wind-dispersed or dispersed by berry-eating birds. Regardless, tree and shrub species richness remained low when compared to natural colonisation sites that are close to diverse seed sources [8, 17]. Coverage of woody vegetation was not uniform between the old fields, with some eventually becoming more heavily wooded than others. This may reflect stochastic variation in seed dispersal and the behaviour of animal or bird dispersers, or variation in growing conditions related to soils or micro-topography, but these variables were not examined.

Noddle Hill is a rare example of natural colonisation progression when conditions are not advantageous for woodland colonisation or development. This contrasts with perceptions of relatively rapid woodland colonisation through passive rewilding, likely resulting from a positive selection bias in the literature for studies of successful woodland establishment [3, 18, 19].

Nevertheless, the results from Noddle Hill tally with established principles of woodland succession on abandoned farmland, albeit at a slower rate, derived from global studies [3, 18]. Prior land use, ground conditions, available seed sources, the presence of seed-dispersing animals and the density of herbivores all drive the successional trajectory of colonising vegetation. Sites with disturbed ground, abundant seed sources and low herbivore density undergo more rapid and extensive natural colonisation by trees and shrubs [3, 8, 18, 46–48].

However, long-term studies of natural colonisation from Western Europe remain uncommon, despite an urgent need to inform policies for woodland expansion and farmland abandonment [4, 24, 49]. In contrast to Noddle Hill, most Western European studies of natural colonisation have been on sites close to abundant seed sources. In lowland England, sites adjacent to woodland or hedgerows had 35–97% canopy cover by shrubs and trees (with bramble as a minority) within 18–40 years, in spite of various herbivores [8, 17, 28]. A study in the Netherlands, however, found that herbivores limited woody colonisation to only 8% of the old field area after 29 years [27]. Similar limitations were also found on wood pasture in Spain [50], but not in Sicily [51].

Despite varied grazing pressure and ground conditions at these European sites, in all cases the colonisation of trees was associated with thorny shrubs, typically bramble or other *Rubus* spp., rose spp., common hawthorn and/or blackthorn. These thorny pioneers, which are primarily dispersed by berry-eating birds (especially thrushes), appear important in sheltering tree seedlings from herbivores [27, 50]. The trees eventually grow to form a woodland canopy over the shrubs, which become the understorey shrub layer [8, 17].

At Noddle Hill, however, where tree colonisation remained very limited, the trajectory of vegetation succession is characterised by an initial and protracted phase of mesotrophic grassland with spontaneous wetlands, followed by a thorny shrubland mosaic. The scarcity of young trees after 33 years suggests that the shrubland phase is likely to persist for many decades.

### Hedgerows and wetlands

Hedgerows are important semi-natural habitats and cultural features in Western Europe, and abandonment is a major cause of hedgerow loss [52]. Noddle Hill’s original hedgerow network became degraded during rewilding, with some loss of length likely due to drowning as the derelict ditches overflowed. Nevertheless, after 33 years much of the hedgerow network was subsumed within the infield natural colonisation. Despite losing cultural value associated with traditional landscapes, hedgerow biodiversity likely benefited from this significant expansion of shrubs onto the old fields.

Wetlands were an important part of the Noddle Hill habitat mosaic, and appeared to have expanded from derelict boundary ditches or developed in infield hollows, probably shortly after abandonment. Wetlands persisted throughout the rewilding period, but did not increase over time or change in vegetation type, and their varying extent may have been due to preceding rainfall or approximate mapping. The wetlands were dominated by dense vegetation, and the habitat mosaic echoed the original fen, swamp and wet woodland (‘carr’) habitat that was regionally common until drainage and clearance in the Medieval period (c.1000 BP) [53, 54].

### Bird community changes, biodiversity and associated ecosystem services

Noddle Hill’s bird community clearly shifted in response to passive rewilding. Farmland birds associated with open habitats declined, but woodland species increased as shrubs and some trees colonised the old fields, despite the fact that the majority of woody cover was low-growing bramble. The loss of some birds from Noddle Hill, and from the BBS squares, was likely a result of national population declines, particularly of farmland specialists, such as grey partridge *Perdix perdix* and corn bunting *Emberiza calandra* [42]. As such, some farmland birds are likely to have been lost from Noddle Hill regardless of habitat changes from rewilding. A few farmland species may have benefited briefly, such as Eurasian skylarks *Alauda arvensis*, which were initially abundant on Noddle Hill’s extensive grassland (an ideal habitat) before disappearing as woody colonisation progressed (S3 Appendix).

The developing habitat mosaic at Noddle Hill resulted in the farmland bird declines being partially mirrored by colonisation of woodland and wetland species, such as Eurasian bullfinch *Pyrrhula pyrrhula*, Eurasian sparrowhawk *Accipiter nisus* and Eurasian teal *Anas crecca*. After 33 years of passive rewilding, the 25 ha of grassland, wetland and shrubland maintained as many bird species as was found in the local farmland BBS squares, but held more woodland and wetland species than in the wider region. Overall, Noddle Hill’s bird community became more locally distinctive (as shown by the Jaccard index).

Rewilding of abandoned land that creates early successional shrubland and wetland mosaics can create refuges for regionally declining birds [5], and Noddle Hill was notable for the abundance of songbirds in this habitat, especially warblers. These included sedge warblers *Acrocephalus schoenobaenus*, common whitethroats *Curruca communis* and willow warblers *Phylloscopus trochilus*, which were scarce in the local farmland and are declining nationally [42, 55]. Shrubland habitats can therefore contribute to conserving some species and enhancing the soundscape of birdsong, which is one of the major ways that people can engage with nature, and improves human wellbeing [56, 57].

These results support the concept of small-scale rewilding as habitat ‘islets’ in farmed landscapes to enhance regional biodiversity and nature recovery [7, 25]. The loss of some farmland habitat specialists and cultural landscape features (hedgerows) at Noddle Hill could be considered minor at the regional level, where farmland remains the dominant land use, and so diversification of the regional habitats was likely more beneficial overall. Other conservation priority species are also likely to have benefitted from Noddle Hill’s rewilding, with species detected during other surveys including great crested newts *Triturus cristatus*, harvest mice *Micromys minutus*, and 16 species of dragonfly (Odonata) [34, 58].

Shrublands are also potentially valuable to pollinators and the ecosystem services they provide. The bramble, common hawthorn and rose spp. that dominated 13 ha of Noddle Hill have a sequential flowering season spanning four months of the year [59, 60]. This pollen and nectar resource is heavily utilised by wild pollinators and domestic honeybees *Apis mellifera* [61, 62], and so shrubland habitats could support important pollination services in the local landscape.

A cultural ecosystem service provided by shrublands is through the prolific berries produced by bramble, rose spp., common elder and/or blackthorn, which are abundant on Noddle Hill and other rewilded shrubland sites [7, 8, 27, 28]. Such berries are collected across Europe and North America for traditional cooking and making beverages [63, 64], including at Noddle Hill (pers. obs.), which is adjacent and accessible to a large urban population. Other recreational uses observed during the study included walking, jogging, birdwatching and photography.

## Conclusions

In line with our main hypotheses, woody colonisation of Noddle Hill was relatively slow, but was associated with a greater shift to woodland birds among the changing bird community. While passive rewilding is often considered in terms of expanding woodland, Noddle Hill is a useful case study of its limitations when local tree cover is low. Despite advantageous ground conditions and few herbivores, there were insufficient seed sources and dispersers nearby to generate rapid succession. The presence of some young trees in 2021 suggests that Noddle Hill will eventually develop areas of closed-canopy woodland, but this may take many more decades.

A major conclusion from our results is that, if woodland cover is a primary goal for sites that are isolated from seed sources, then realising closed-canopy woodland within a reasonable timeframe would require active restoration by planting. However, in terms of nature recovery, Noddle Hill was successful in achieving habitat diversification in the wider agricultural landscape and enhancing or maintaining regional biodiversity. The shrubland habitat mosaic, with areas of open grassland and small wetlands, provided important habitats for birds, including nationally declining songbirds and wetland species that were otherwise scarce in the wider region. Other taxa are similarly likely to benefit from the creation of shrubland habitat mosaics via planned or accidental rewilding, even if closed-canopy woodland does not rapidly develop.

As such, the core findings from Noddle Hill are that the slow natural colonisation and vegetation succession, dominated by blossom- and berry-rich thorny shrubs, could provide important ecosystem services of enhanced biodiversity, pollinator resources and cultural services for many decades before any closed-canopy woodland develops. Consequently, shrublands and any associated wetlands can be recognised as important habitats in policies aimed at promoting natural colonisation for nature recovery. Shrubland mosaics may form a valuable transitional stage in the succession to closed-canopy woodland, including protection from herbivores for growing trees. Alternatively, establishment of long-lasting shrublands may represent restoration of a habitat with its own intrinsic ecological and social value. Both eventualities could be viewed as successful and valid outcomes within a wider woodland and habitats strategy, with their own range of benefits for nature and society.

## Supporting information

S1 Appendix Details of lidar data acquisition, processing and availability. (DOCX)

S2 Appendix Vegetation communities recorded on Noddle Hill in 2001. (DOCX)

S3 Appendix Bird counts at Noddle Hill and in Breeding Bird Survey (BBS) squares. (DOCX)

S1 Dataset Compressed/ZIP file archive of raster geoTiff GIS data for the Noddle Hill DSM in 2021. (ZIP)
