# Supplementary material for: Slow development of woodland vegetation and bird communities during 33 years of passive rewilding in open farmland
Source: PLoS One. 2022 Nov 11;17(11):e0277545. doi: 10.1371/journal.pone.0277545 (PMC9651571; doi:10.1371/journal.pone.0277545)
Supplement: S1 Appendix — (DOCX) [file pone.0277545.s001.docx]

**S1 Appendix.** Details of lidar data acquisition and processing

The lidar datasets for this study were collected during 2002, 2008 and 2021. The first two years involved lidar data products collected and processed by England’s Environment Agency, derived from a lidar sensor mounted on a fixed-wing aircraft. In 2021, the lidar was collected and processed by the UK Centre for Ecology & Hydrology, using a sensor mounted on a UAV (drone).

The lidar scanners varied in their acquisition parameters (Table S1). Environment Agency lidar data were processed using standard methods to classify pulse returns as ground or above-ground (Axelsson 2000), and quality control was applied (using ground control points) to generate raster products with a vertical accuracy of ±0.15 m or better. Six ground control points were used, with an RSME of 0.05-0.08. The output lidar products are available for public download via a dedicated platform as a Digital Terrain Model (DTM) or Digital Surface Model (DSM) (see below for data availability).

Environment Agency (EA) data were collected during leaf-off (winter) conditions, when deciduous trees and shrubs would have been less detectable. First returns of the lidar pulse were filtered out during processing, meaning that the DSM does not depict the maximum canopy height of vegetation. Instead, the DSM can detect the presence of above-ground vegetation, but not accurate heights.

UKCEH data processing details

Further lidar data were collected by the UK Centre for Ecology & Hydrology (UKCEH) during leaf-on conditions (autumn) when deciduous shrubs and trees were in full leaf. A DJI M300 RTK drone fitted with a DJI Zenmuse L1 Lidar scanner was flown over the study area. The flight was conducted at 80 m altitude with a 20% overlap using triple returns and a sampling frequency of160 K/s. The IMU was calibrated every 100 seconds. An EMLID Reach RS+ Differential GPS base station was located within the study area to provide maximum precision, and all data were post processed using PPK in DJI Terra software. The resulting point cloud was then processed in Terrasolid’s Terrascan software to remove outliers and generate the DSM at 0.2 m resolution.

The DSM and DTM data were used to create Canopy Height Models (CHM). A CHM is a raster surface created by subtracting the DTM (ground returns) from the DSM (surface returns).

Table S1. Acquisition parameters of the different lidar datasets used in the study

| **Acquisition parameter** | **Year** | | |
| --- | --- | --- | --- |
|  | **2002** | **2008** | **2021** |
| Source | EA | EA | UKCEH |
| Platform | Airplane | Airplane Cessna 404 | UAV |
| Scanner | Optech ALTM-2033 | Optech ALTM-3100 | DJI L1 |
| Flying altitude (agl) | 900 m | 800 m | 50 m |
| Flying date | 05/12/2002 | 22/01/2008 | 28/09/2021 |
| Max. no. of registered returns per pulse | 4 | 4 | 3 |
| Average pulse density | c. 0.2/m^2^ | 4/m^2^ | 400/m^2^ |
| Processed resolution | 2 m | 0.5 m | 0.2 m |

Reference

Axelsson P. DEM generation from laser scanner data using adaptive TIN models. Int. Arch. Photogramm. Remote Sens. 2000; 33: 110-117.

Data availability

The Environment Agency lidar data products are available from the following location: https://environment.data.gov.uk/DefraDataDownload/?Mode=survey

Filenames:

**2002 DTM:** DTM_F0023991_20021205_20021205

**2002 DSM:** DSM_D0023991_20021205_20021205

**2008 DTM:** DTM_F0091674_20080122_20080207, DTM_F0091686_20080122_20080207, DTM_F0091673_20080122_20080207, DTM_F0091685_20080122_20080207,

The UKCEH lidar DSM for 2021 is supplied as a geoTIFF in the folder S1 Dataset.
