# Supplementary material for: Slow development of woodland vegetation and bird communities during 33 years of passive rewilding in open farmland
Source: PLoS One. 2022 Nov 11;17(11):e0277545. doi: 10.1371/journal.pone.0277545 (PMC9651571; doi:10.1371/journal.pone.0277545)
Supplement: S2 Appendix — (DOCX) [file pone.0277545.s002.docx]

**S2 Appendix.** Vegetation communities recorded by Holloway (2001) on Noddle Hill in 2001, after 13 years of passive rewilding. Codes refer to the UK National Vegetation Classification (NVC) as defined in Rodwell (1992) and Rodwell (2000).

Mesotrophic grassland communities:

MG1 *Arrhenatherum elatius* grassland – dominant on site,

MG6 *Lolium perenne*–*Cynosurus cristatus* grassland – common.

MG9 *Holcus lanatus*–*Deschampsia cespitosa* grassland – common.

MG11 *Festuca rubra*–*Agrostris stolonifera*–*Potentilla anserine* grassland – present.

Vegetation of open habitats:

OV21 *Poa annua*–*Plantago major* community – present

OV23 *Lolium perenne*–*Dactylis glomerata* community – dominant

OV29 *Alopecurus geniculatus*–*Rorippa palustris* community – present

**References**

Holloway S. BGEEP Biodiversity Project North Carr, Bransholme 2001. Bishop Burton College: Bishop Burton; 2001.

Rodwell JS (ed.). British Plant Communities. Volume 3. Grassland and Montane Communities. Cambridge University Press: Cambridge; 1992.

Rodwell JS (ed.). British Plant Communities. Volume 5. Maritime communities and Vegetation of Open Habitats. Cambridge University Press: Cambridge; 2000.
