# Supplementary material for: Slow development of woodland vegetation and bird communities during 33 years of passive rewilding in open farmland
Source: PLoS One. 2022 Nov 11;17(11):e0277545. doi: 10.1371/journal.pone.0277545 (PMC9651571; doi:10.1371/journal.pone.0277545)
Supplement: S3 Appendix — (DOCX) [file pone.0277545.s003.docx]

**S3 Appendix**. Maximum counts from transect surveys of breeding birds at Noddle Hill (NH) and British Trust for Ornithology Breeding Bird Survey (BBS) counts in 1 km squares within 10 km of NH, with five individual squares identified as BBS1 to BBS5. Surveys took place in three survey periods in the specified years. Asterisk (*) denotes 21 species of songbird (warbler, finch, bunting and thrush) where maximum counts from NH and the BBS squares were compared between in Period 3, using a Wilcoxon signed rank test (*W* = 5.5 *P* = 0.001).

| **English name** | **Scientific name** | **Habitat association** | **Period 1** | | | **Period 2** | | | **Period 3** | | |
| --- | --- | --- | --- | --- | --- | --- | --- | --- | --- | --- | --- |
|  |  |  | **1992** | **1994** | | **2006** | | | **2021** | **2017** | |
|  |  |  | **NH** | **BBS1** | **BBS2** | **NH** | **BBS3** | **BBS2** | **NH** | **BBS4** | **BBS5** |
| Eurasian Sparrowhawk | *Accipiter nisus* | Woodland | 0 | 1 | 0 | 2 | 0 | 1 | 1 | 0 | 0 |
| Sedge Warbler* | *Acrocephalus schoenobaenus* | Wetland | 17 | 0 | 0 | 9 | 0 | 0 | 13 | 0 | 0 |
| Reed Warbler* | *Acrocephalus scirpaceus* | Wetland | 1 | 0 | 0 | 7 | 0 | 0 | 3 | 0 | 0 |
| Long-tailed Tit | *Aegithalos caudatus* | Woodland | 1 | 0 | 0 | 0 | 0 | 0 | 3 | 1 | 1 |
| Eurasian Skylark | *Alauda arvensis* | Farmland | 20 | 6 | 2 | 11 | 8 | 2 | 0 | 0 | 5 |
| Common Kingfisher | *Alcedo atthis* | Wetland | 1 | 0 | 0 | 1 | 0 | 0 | 0 | 0 | 0 |
| Red-legged Partridge | *Alectoris rufa* | Farmland | 0 | 2 | 0 | 0 | 2 | 2 | 0 | 0 | 0 |
| Eurasian Teal | *Anas crecca* | Wetland | 0 | 0 | 0 | 6 | 0 | 0 | 2 | 0 | 0 |
| Mallard | *Anas platyrhynchos* | Wetland | 4 | 0 | 6 | 25 | 1 | 5 | 11 | 0 | 3 |
| Greylag Goose | *Anser anser* | Wetland | 0 | 0 | 0 | 45 | 2 | 1 | 3 | 0 | 0 |
| Meadow Pipit | *Anthus trivialis* | Farmland | 8 | 2 | 0 | 0 | 2 | 3 | 0 | 0 | 0 |
| Common Swift | *Apus apus* | n/a | 0 | 1 | 0 | 0 | 0 | 0 | 1 | 4 | 8 |
| Grey Heron | *Ardea cinerea* | Wetland | 1 | 1 | 0 | 0 | 0 | 0 | 0 | 0 | 1 |
| Canada Goose | *Branta canadensis* | Wetland | 0 | 0 | 0 | 0 | 3 | 0 | 0 | 0 | 0 |
| Common Buzzard | *Buteo buteo* | Woodland | 0 | 0 | 0 | 0 | 0 | 0 | 1 | 1 | 1 |
| European Goldfinch* | *Carduelis carduelis* | Farmland | 2 | 0 | 0 | 4 | 2 | 3 | 10 | 3 | 24 |
| Cetti's Warbler* | *Cettia cetti* | Wetland | 0 | 0 | 0 | 0 | 0 | 0 | 4 | 0 | 0 |
| European Greenfinch* | *Chloris chloris* | Farmland | 3 | 0 | 2 | 7 | 2 | 4 | 1 | 0 | 9 |
| Marsh Harrier | *Circus aeruginosus* | Wetland | 0 | 0 | 0 | 0 | 0 | 0 | 1 | 0 | 0 |
| Stock Dove | *Columba oenas* | Farmland | 8 | 0 | 0 | 3 | 4 | 13 | 2 | 0 | 2 |
| Woodpigeon | *Columba palumbus* | Farmland | 35 | 9 | 10 | 65 | 8 | 17 | 42 | 9 | 27 |
| Carrion Crow | *Corvus corone* | Farmland | 2 | 3 | 3 | 7 | 4 | 8 | 16 | 2 | 11 |
| Rook | *Corvus frugilegus* | Farmland | 4 | 2 | 1 | 4 | 10 | 0 | 0 | 8 | 0 |
| Jackdaw | *Corvus monedula* | Farmland | 3 | 0 | 0 | 0 | 3 | 1 | 2 | 21 | 0 |
| Common Cuckoo | *Cuculus canorus* | Wetland | 1 | 3 | 1 | 2 | 1 | 1 | 0 | 0 | 0 |
| Common Whitethroat* | *Curruca communis* | Farmland | 13 | 2 | 0 | 14 | 2 | 4 | 18 | 0 | 3 |
| Lesser Whitethroat* | *Curruca curruca* | Woodland | 2 | 1 | 0 | 1 | 0 | 1 | 6 | 0 | 0 |
| Blue Tit | *Cyanistes caeruleus* | Woodland | 12 | 3 | 1 | 9 | 2 | 3 | 4 | 3 | 3 |
| Mute Swan | *Cygnus olor* | Wetland | 1 | 0 | 0 | 2 | 0 | 0 | 3 | 0 | 0 |
| House Martin | *Delichon urbica* | Farmland | 15 | 0 | 1 | 4 | 0 | 2 | 0 | 4 | 1 |
| Great Spotted Woodpecker | *Dendrocopos major* | Woodland | 0 | 0 | 0 | 0 | 0 | 0 | 0 | 1 | 0 |
| Corn Bunting* | *Emberiza calandra* | Farmland | 0 | 2 | 0 | 0 | 0 | 1 | 0 | 0 | 0 |
| Yellowhammer* | *Emberiza citrinella* | Farmland | 4 | 5 | 0 | 2 | 2 | 3 | 0 | 0 | 2 |
| Reed Bunting* | *Emeriza schoeniclus* | Farmland | 5 | 2 | 2 | 14 | 2 | 0 | 3 | 0 | 1 |
| European Robin | *Erithacus rubecula* | Woodland | 4 | 2 | 1 | 5 | 1 | 3 | 5 | 4 | 4 |
| Eurasian Kestrel | *Falco tinnunculus* | Farmland | 2 | 0 | 0 | 1 | 0 | 1 | 1 | 0 | 1 |
| Common Chaffinch* | *Fringilla coelebs* | Woodland | 3 | 1 | 0 | 5 | 3 | 2 | 5 | 8 | 3 |
| Eurasian Coot | *Fulicra atra* | Wetland | 0 | 0 | 0 | 8 | 0 | 0 | 1 | 0 | 0 |
| Common Snipe | *Gallinago gallinago* | Wetland | 1 | 0 | 0 | 1 | 0 | 0 | 0 | 0 | 0 |
| Common Moorhen | *Gallinula chloropus* | Wetland | 7 | 0 | 1 | 7 | 1 | 1 | 7 | 0 | 0 |
| Barn Swallow | *Hirundo rustica* | Farmland | 5 | 8 | 2 | 2 | 10 | 8 | 1 | 1 | 6 |
| Common Linnet* | *Linaria cannabina* | Farmland | 10 | 11 | 1 | 10 | 6 | 8 | 31 | 4 | 13 |
| Grasshopper Warbler* | *Lucustella naevia* | Wetland | 1 | 0 | 0 | 1 | 0 | 0 | 0 | 0 | 0 |
| Pied/White Wagtail | *Motacilla alba* | Farmland | 2 | 2 | 0 | 0 | 0 | 3 | 0 | 0 | 1 |
| Yellow Wagtail | *Motacilla flava* | Farmland | 1 | 1 | 0 | 0 | 1 | 1 | 0 | 0 | 0 |
| Great Tit | *Parus major* | Woodland | 6 | 1 | 1 | 13 | 3 | 4 | 3 | 4 | 3 |
| House Sparrow | *Passer domesticus* | Farmland | 0 | 5 | 12 | 4 | 8 | 7 | 0 | 1 | 7 |
| Tree Sparrow | *Passer montanus* | Farmland | 1 | 8 | 0 | 0 | 0 | 5 | 0 | 2 | 2 |
| Grey Partridge | *Perdix perdix* | Farmland | 6 | 0 | 2 | 0 | 2 | 3 | 0 | 0 | 0 |
| Common Pheasant | *Phasianus colchicus* | Farmland | 5 | 1 | 1 | 8 | 2 | 4 | 2 | 1 | 3 |
| Common Chiffchaff* | *Phylloscopus collybita* | Woodland | 0 | 0 | 0 | 1 | 0 | 0 | 8 | 1 | 2 |
| Willow Warbler* | *Phylloscopus trochilus* | Woodland | 3 | 1 | 0 | 8 | 1 | 0 | 11 | 0 | 0 |
| Eurasian Magpie | *Pica pica* | Farmland | 4 | 0 | 0 | 5 | 2 | 4 | 5 | 2 | 7 |
| Green Woodpecker | *Picus viridis* | Woodland | 0 | 1 | 0 | 0 | 0 | 0 | 0 | 0 | 0 |
| Marsh Tit | *Poecile palustris* | Woodland | 0 | 0 | 0 | 0 | 0 | 1 | 0 | 0 | 0 |
| Dunnock | *Prunella modularis* | Woodland | 8 | 3 | 2 | 8 | 3 | 6 | 5 | 2 | 2 |
| Eurasian Bullfinch* | *Pyrrhula pyrrhula* | Woodland | 0 | 0 | 0 | 0 | 0 | 0 | 4 | 0 | 0 |
| Water Rail | *Rallus aquaticus* | Wetland | 0 | 0 | 0 | 0 | 0 | 0 | 1 | 0 | 0 |
| Collared Dove | *Streptopelia decaocto* | Farmland | 1 | 1 | 3 | 3 | 0 | 5 | 1 | 1 | 2 |
| Turtle Dove | *Streptopelia turtur* | Farmland | 2 | 0 | 0 | 0 | 0 | 0 | 0 | 0 | 0 |
| Common Starling | *Sturnus vulgaris* | Farmland | 70 | 4 | 16 | 46 | 11 | 11 | 50 | 30 | 4 |
| Eurasian Blackcap* | *Sylvia atricapilla* | Woodland | 0 | 0 | 0 | 2 | 0 | 0 | 2 | 2 | 2 |
| Garden Warbler* | *Sylvia borin* | Woodland | 0 | 0 | 0 | 0 | 1 | 1 | 1 | 0 | 0 |
| Shelduck | *Tadorna tadorna* | Wetland | 0 | 0 | 0 | 0 | 0 | 4 | 0 | 0 | 0 |
| Winter Wren | *Troglodytes troglodytes* | Woodland | 6 | 4 | 2 | 14 | 1 | 5 | 20 | 2 | 5 |
| Common Blackbird* | *Turdus merula* | Woodland | 10 | 4 | 9 | 14 | 5 | 14 | 21 | 10 | 13 |
| Song Thrush* | *Turdus philomelos* | Woodland | 5 | 0 | 3 | 5 | 4 | 2 | 7 | 2 | 3 |
| Mistle Thrush* | *Turdus viscivorus* | Farmland | 0 | 0 | 0 | 0 | 0 | 0 | 0 | 0 | 1 |
| Barn Owl | *Tyto alba* | Farmland | 1 | 0 | 0 | 3 | 0 | 1 | 0 | 1 | 1 |
| Northern Lapwing | *Vanellus vanellus* | Farmland | 6 | 0 | 0 | 2 | 4 | 0 | 0 | 0 | 1 |
